# University hospitals as drivers of career success: an empirical study of the duration of promotion and promotion success of hospital physicians

**DOI:** 10.1186/1472-6920-14-85

**Published:** 2014-04-23

**Authors:** Christiane Degen, Ludwig Kuntz

**Affiliations:** 1 Department of Business Administration and Health Care Management, University of Cologne, Albertus-Magnus-Platz, Cologne 50931, Germany

**Keywords:** Career success, Hospital physicians, Promotion, University hospitals

## Abstract

**Background:**

German hospitals have a well-defined career structure for clinicians. In this hierarchical career system university hospital are stepping stones for career advancement. This longitudinal study investigates the impact of working in university hospitals on the career success of junior physicians and senior physicians.

**Methods:**

Consideration of the career trajectories of 324 hospital physicians. Discrete-time event history analysis is used to study the influence of working in university hospitals on the chance of promotion from junior physician to senior physician and senior physician to chief physician. A comparison of medians provides information about the impact of working in university hospitals on the duration of promotion to senior and chief physician positions.

**Results:**

Working in university hospitals has a negative impact for advancement to a senior physician position in terms of promotion duration (p = 0.005) and also in terms of promotion success, where a short time span of just 1–2 years in university hospitals has a negative effect (OR = 0.38, p < 0.01), while working there for a medium or long term has no significant effect. However, working in universities has a positive effect on the duration of promotion to a chief physician position (p = 0.079), and working in university hospitals for 3–4 years increases the chance of promotion to a chief physician position (OR = 4.02, p < 0.05), while working there > =7 years decreases this chance (OR = 0.27, p < 0.05). In addition, physicians have a higher chance of promotion to a chief physician position through career mobility when they come to the position from a university hospital.

**Conclusion:**

Working at university hospitals has a career-enhancing effect for a senior physician with ambitions to become a chief physician. For junior physicians on the trajectory to a senior physician position, however, university hospitals are not drivers of career success.

## Background

Physicians in Germany have three options when choosing a career path in medicine: they can go into private practice, become a hospital physician, or follow an academic route. Physicians who aspire to advance in the hospital career system move up the hierarchical ladder from a junior physician position to that of a senior physician, and from there to the position of chief physician of a department. Junior doctors are residents that have a licence to practice medicine and are in training for a medical specialty [1,2]. The job profile of a senior physician assumes medical responsibility for sub-domains and demands a high level of professional qualification, as well as management ability [1]. In the chief physician post the requirements in the areas of medicine and management are combined with leadership skills, scientific reputation, and the ability to lead a department strategically within the overall structure of the hospital [1,3,4]. The career development of physicians begins upon completion of study and takes the form of training in clinical practice, which is influenced by the organizational and personnel-related factors of a particular hospital [5]. Hospital-based physicians working in in-patient care can be found in many countries (e.g. the USA (hospitalists) [6], Norway (hospital physicians) [7], the UK (hospital consultants) [8]).

Arguments for why working in a university hospital prepares a physician for advancement in the so-called ‘chief physician system’ are largely based on the high-performance medicine practised in such institutions, the multiple qualification options in the clinical field, and the research-friendly environment [9]. Moreover, in Germany and countries such as Switzerland and Austria almost all clinical physicians that hold chief physician positions are also successful researchers [10]. Arguments for why working in a university hospital may delay the career development of a junior physician are poor structures for training and supervision. Studies in different country settings on the perceived quality of supervision and professional training by registrars and specialist registrars found that non-university hospitals offered better quality supervision and training than university hospitals [11-13]. Large university hospitals tend to have complex structures and increased interfaces as a result of their high degree of specialization in multiple departments [14], under which the career requirements of individual physicians may suffer. However, a study from the US has shown that large-scale hospitals provide greater freedom for career and research activities due to the possibility of patient hand-offs [15]. Furthermore, training and working in a university hospital is rated highly by German physicians. With regard to filling vacant positions, an experienced senior physician from a prestigious university hospital is often said to be the ideal candidate for a chief physician position [16].

Previous studies on the career success of clinical physicians have focussed on researching physicians’ subjective career success in terms of the outcome variable ‘career satisfaction’ [17-20]. Studies on the objective career success of clinical physicians, which are analysed in the career research literature in terms of hierarchy level achieved, salary, or speed of career progression [21-23] have only been conducted for the outcome variable ‘salary progression’ [24]. The objective of this study is to fill this gap in the research by conducting an investigation into objective measures of advancement in terms of hierarchy level and duration of promotion. A further objective of this quantitative longitudinal study is the investigation of the relationship between working in university hospitals vs non-university hospitals and objective career success in a hierarchical career system. To achieve this, the influence of working in a university hospital on the duration of promotion and promotion success is researched separately for the career movements of junior physicians and senior physicians.

## Methods

### Sample

The investigation is based on a Germany-wide longitudinal dataset of hospital physicians. The data was collected from the candidate database of a personnel consultancy firm specializing in the health-care sector. The permission to use the data was obtained by the personnel consultancy which provided us with the data set for research purposes. The data contains the career and education histories of 324 physicians, from which an average of 17.55 years can be investigated for each professional for the period 1968–2008. During this period, the physicians worked in a total of 596 hospitals, which includes all 32 German university teaching hospitals. Of the 324 junior physicians in the dataset, 310 were observed until promotion to a senior physician position and 177 until promotion to a chief physician position. An overview of selected characteristics of the sample is shown in Table 1.

**Table 1:** Characteristics of physicians examined

**Characteristic**	**Number (%)**	**Mean**	**SD**
Gender			
Female	20 (6.17)		
Male	304 (93.83)		
Specialty			
Anaesthesia	42 (12.96)		
Surgery	118 (36.42)		
Gynaecology	32 (23.15)		
Internal medicine	75 (9.98)		
Paediatrics	16 (4.94)		
Other specialty	41 (12.65)		
Physicians with a scientific degree	79 (24.38)		
Age (years) at			
Career start		28.61	2.84
Promotion to senior physician		36.74	3.83
Promotion to chief physician		44.59	4.79
Inter-organizational moves		2.70	1.63

### Statistical analysis and methods

Discrete-time event history analysis is applied for the investigation of promotion success. In order to fit the data to our discrete-time event history model the data were organized into a longitudinal person-period dataset. This makes it possible to consider records that do not experience the interesting event (e.g., promotion to a chief physician position) and estimate the results using a logistic regression [25]. For the analysis of the initial career stage from junior physician to senior physician, 3,024 physician-years were available, while 2,971 physician-years were considered for the second career stage from senior physician to chief physician. The impact of working in a university hospital – compared to not working in a university hospital – on the chance of promotion was predicted with different regression models for both career stages. The analysis of the duration of promotion was performed by a median-comparison test as the variance is not equal across the samples. We examined the duration of promotion from junior physician to senior physician and senior physician to chief physician for different group variables.

### Study variables

In the following all study variables are described. For the time-dependent variables we also include an overview in Table 2.

**Table 2:** Overview of construction and rationale for inclusion of time dependent variables in the analysis of promotion

**Time-dependent variable**	**Means of construction**	**Reason for inclusion**
**University hospital explanatory variables**		
University hospital yes/no (previous year)	One-year lagged variable that is 1 for working in a university hospital and 0 for all other hospitals.	To examine the influence of working in a university hospital in the previous year on the chance of promotion.
Time in university hospitals at rank	Begins with 0 and counts the cumulated number of years working in university hospitals at a particular rank.	To examine the impact of work experience in university hospitals on the chance of promotion.
Time in university hospitals at rank differentiated into 1–2 years, 3–4 years, 5–6 years, and > =7 years	Constructed from *time in university hospitals at rank*; four variables that switch from 0 to 1 in the first, third, fourth, and seventh years of working in a university hospital.	To gain a deeper understanding of the impact of short-term, mid-term, and long-term work experience in university hospitals on the chance of promotion.
**Career stage-specific control variables**		
Time in rank	Starts with 1 and increases by 1 with each year in rank.	To examine the influence of occupational experience on the chance of promotion.
Time in rank squared	Squared variable of *time in rank*.	In connection with the variable *time in rank*, this variable models the growth of occupational experience and takes into account that the growth rate will decline over time.
Doctorate in medicine	Variable that switches from 0 to 1 the year a doctorate in medicine is obtained.	To examine the impact of obtaining a PhD in medicine on the chance of promotion.
Scientific degree	Variable that switches from 0 to 1 the year a habilitation is obtained.	To examine the impact of attaining a habilitation in medicine (highest scientific degree) on the chance of promotion.
Time to medical specialist	Starts with 1 and increases by 1 each year, remaining constant after the speciality is completed.	To examine the impact of the fast attainment of a medical specialization on the chance of promotion.
Medical specialty completed	Variable that switches from 0 to 1 the year a medical specialty is completed.	Completion of a medical speciality is compulsory for promotion from junior to senior physician. The variable indicates the effect of this occupational demand on the chance of promotion.
**General control variables**		
Previous hospital tenure	Presents the total tenure at the previous hospital. The variable is constant for each year in the current hospital.	In connection with the movement variables, this variable controls for continuity with previous employers. It allows us to examine the impact of a longer tenure with the previous employer on the chance of promotion.
History of inter-organizational moves	Starts with 0 and increases by 1 for each change between hospitals.	To examine the influence of all employer changes over the course of a career on the chance of promotion.
Inter-organizational move	Variable that is 0 in years with no inter-organizational move and 1 in years with an inter-organizational move.	To examine the impact of an inter-organizational move on the chance of promotion.

#### University hospital as an explanatory variable

The influence of working in a university hospital on career success was investigated using different time-dependent and time-independent variables. *University hospital yes/no (previous year)* is a one year-lagged time-dependent variable that has the value 1 for physicians working in a university hospital and 0 for all other hospitals. With this lagged variable it is possible to examine the influence of working in a university hospital in the previous year on the chance of promotion. The time span variables (*total time span* and *during time span*) for working in a university hospital are dummy variables. For example, the variable *total time span* is 1 (otherwise 0) if a physician works in the respective position at one or more university hospitals for the entire time period. *Time in university hospital at rank* is a time-dependent variable that presents the cumulated number of years working in university hospitals on a hierarchical level. A further time differentiation is made by four time-dependent variables (*1–2 years*, *3–4 years*, *5–6 years*, and *> =7 years*), which switch from 0 to 1 in the first, third, fourth, and seventh years of working in a university hospital.

#### Career stage-specific control variables

Important determinants for the measurement of career success on a career stage are occupational experience in the rank and career stage-specific qualifications [26]. Occupational experience is modelled with a time-dependent variable *time in rank* for each hierarchy level. A squared variable (*time in rank squared*) is also used as the growth of occupational experience in a career stage does not show a continuing linear increase. A positive value for *time in rank* and a negative value for the *time in rank squared* variable indicate concave growth in occupational experience. Different time-dependent variables are used for professional qualification according to the hierarchy level. These variables switch from 0 to 1 at the point when the qualification is earned. For promotion to senior physician these are the variables *doctorate in medicine*, *scientific degree*, and *medical specialty completed*. For promotion to chief physician this is the variable *scientific degree*; this is measured by the attainment of habilitation, which is a formal postdoctoral qualification in research. *Time to medical specialist* increases by one each year and remains constant after the speciality is obtained. To control for possible differences in career trajectories between medical specialties in the analysis of promotion to a chief physician position, dummy variables are used for anaesthesia, surgery, gynaecology, paediatrics, and ‘other’ specialties, and internal medicine is taken as a reference category. *Age at career start* is used in the analysis of promotion to a senior physician position (and *age at middle career stage start* for promotion to a chief physician position) as a time-constant variable to control for physicians with differing time frames for career progression. *Time to mid-level position* is constant and controls for the speed of career progression up to that point.

#### General control variables

*Gender* is used as a control variable with the value 1 for male physicians and 0 for female physicians. The variable *previous hospital tenure* is a time-dependent covariate that indicates the total tenure at the previous hospital for each year in the present hospital. A further determinant of career success is inter-organizational mobility [27,28]. The variable *history of inter-organizational moves* is a time-dependent variable that presents the cumulated number of changes between hospitals. With this variable it is possible to analyse the influence of employer changes over the course of a career. For this purpose the variable is lagged by one year to eliminate the possible effect of a direct move to a senior physician or chief physician position. *Inter-organizational move* is a time-dependent variable with value 1 whenever an individual in the dataset changes from one hospital to another and 0 otherwise, indicating the probability of rank advancement through an inter-organizational move in the period that the move takes place. As inter-organizational mobility is an important determinant of career success, we also created an interaction of this move variable and the explanatory variable *university hospital yes/no (previous year)*. The interaction controls for the effect of changing from a university hospital to another hospital on the chance of promotion.

## Results

### Promotion duration and university hospitals

Two different time span concepts were chosen for the examination of the duration of the time period leading up to promotion. The upper section of Table 3 shows the sample differentiate between a group of physicians working for the entire period as either a junior or senior physician in a university hospital and a group not working in such an institution for the whole time. The median comparison indicates that senior physicians spending the total time period in university hospitals obtain a chief physician position faster than those with either no time or only a partial time period spent in university hospitals. As this result is only marginally significant (p = 0.079) we also examined the survival functions for these two groups and found that the function of senior physicians with the total time span in university hospitals was always under the function of the other group. This further confirms the earlier result. By comparison, there is no differentiation for junior physicians between these two groups for the promotion period leading up to a senior physician position.

**Table 3:** Comparison of medians for duration in university hospitals vs non-university hospitals differentiated by hierarchy stage

**… in university hospital**	**Senior physician**		**Chief physician**	
	**Number (N = 310)**	**Median duration from junior to senior physician (years)**	**Number (N = 177)**	**Median duration from senior to chief physician (years)**
**Total time span …**	64	7.5	53	6
**Partial or no time span …**	246	8	124	8
**Chi-squared test**		**p = 0.910**		**p = 0.079**
**During time span …**	151	8	69	7
**Not during time span …**	159	7	108	7.5
**Chi-squared test**		**p = 0.005**		**p = 0.925**

The second time span concept divides the sample into physicians who worked as a junior or senior physician in a university hospital during the time span and those not working there over the time span (lower section of Table 3). In this assessment there is no difference in the time period spent reaching a chief physician promotion. However, junior physicians who worked in university hospitals for some of the time period took longer to gain promotion to a senior physician position than junior physicians with no work experience in university hospitals. To rule out the possibility that a longer duration for promotion to a senior physician post is based on the more frequent hospital changes of the 151 junior physicians in comparison to the 159 physicians in the reference group, another median-comparison test was carried out for the number of hospitals changes. The test result was not significant.

Additionally, survival curves for years from junior to senior physician and senior to chief physician were plotted by >=1, >=3, >=5 and >=7 years in university hospitals. These graphs also confirm that junior physicians in university hospitals take longer to achieve promotion, whereas senior physicians in university hospitals attain a chief physician position faster. Survival curves are shown in Additional files 1 and 2.

### Promotion success and university hospitals

The results of the regression analysis for promotion to senior physician are shown in Table 4 and to chief physician in Table 5.

**Table 4:** Logistic regression for promotion from junior physician to senior physician

		**Model 1**	**Model 2**	**Model 3**
		**OR (95% CI)**	**OR (95% CI)**	**OR (95% CI)**
**Career stage-specific control variables**				
Time in rank		1.49*** (1.19–1.86)	1.46*** (1.16–1.83)	1.50*** (1.20–1.88)
Time in rank squared		0.98*** (0.97–0.99)	0.98*** (0.97–0.99)	0.98*** (0.97–0.99)
Age at career start		1.00 (0.94–1.06)	0.99 (0.93–1.05)	1.00 (0.94–1.06)
Doctorate in medicine		1.50 (0.96–2.33)	1.51 (0.96–2.36)	1.45 (0.93–2.26)
Scientific degree		2.57* (1.16–5.72)	2.68* (1.19–6.03)	2.52* (1.15–5.53)
Time to medical specialist		1.14** (1.03–1.25)	1.13* (1.03–1.24)	1.13** (1.03–1.24)
Medical specialty completed		18.11*** (10.89–30.11)	18.04*** (10.86–29.96)	17.87*** (10.76–29.74)
**General control variables**				
Previous hospital tenure		0.96 (0.91–1.03)	0.97 (0.91–1.03)	0.96 (0.90–1.03)
Inter-organizational move		12.35*** (8.47–17.98)	13.39*** (9.12–19.68)	12.88*** (8.37–19.80)
History of inter-organizational moves		0.93 (0.80–1.07)	0.98 (0.84–1.15)	0.93 (0.80–1.07)
Gender^1^		1.32 (0.71–2.45)	1.40 (0.74–2.64)	1.31 (0.70–2.44)
**Explanatory variables**				
Time in university hospital at rank Time in university hospital at rank		1.02 (0.98–1.06)		
1–2 years			0.38** (0.21–0.70)	
3–4 years			1.83 (0.84–3.96)	
5–6 years			1.73 (0.84–3.58)	
> = 7 years			0.90 (0.49–1.62)	
University hospital yes/no (previous year)				1.37 (0.92–2.03)
Interaction^2^				0.96 (0.49–1.87)
**Observations**		3,024	3,024	3,024
**Pseudo R-squared**		0.423	0.430	0.425
**Log likelihood**		-577.6	-571.3	-576.4

^1^Reference category = female.

^2^Interaction of *inter-organizational move* and *university hospital yes/no (previous year).*

***p <0.001; **p <0.01; *p <0.05.

**Table 5:** Logistic regression for promotion from senior physician to chief physician

		**Model 1**	**Model 2**	**Model 3**
		**OR (95% CI)**	**OR (95% CI)**	**OR (95% CI)**
**Career stage–specific control variables**				
Time in rank		1.60*** (1.37–1.88)	1.59*** (1.35–1.89)	1.61*** (1.36–1.91)
Time in rank squared		0.99*** (0.98–0.99)	0.99*** (0.98–0.99)	0.99*** (0.98–0.99)
Time to mid-level position		0.88* (0.80–0.97)	0.89* (0.81–0.98)	0.89* (0.81–0.98)
Age at middle career stage start		1.03*** (1.01–1.04)	1.03*** (1.02–1.04)	1.03*** (1.01–1.04)
Scientific degree		6.80*** (3.39–13.62)	6.13*** (2.98–12.63)	7.25*** (3.62–14.54)
Anaesthesia^1^		0.96 (0.42–2.20)	0.88 (0.38–2.04)	0.93 (0.41–2.12)
Surgery^1^		0.91 (0.51–1.63)	0.79 (0.44–1.43)	0.92 (0.52–1.65)
Gynaecology^1^		0.70 (0.30–1.60)	0.67 (0.29–1.54)	0.78 (0.33–1.84)
Paedatrics^1^		1.08 (0.32–3.64)	1.00 (0.28–3.55)	1.16 (0.34–3.98)
Other specialty^1^		0.86 (0.36–2.06)	0.82 (0.34–2.00)	0.91 (0.38–2.18)
**General control variables**				
Previous hospital tenure		1.13*** (1.06–1.22)	1.13*** (1.05–1.21)	1.11** (1.03–1.19)
Inter-organizational move		159.83*** (84.42–302.61)	175.01*** (91.01–336.54)	115.97*** (60.59–222.00)
History of inter-organizational moves		1.30** (1.06–1.58)	1.29* (1.06–1.58)	1.26* (1.04–1.54)
Gender^2^		1.92 (0.52–7.04)	2.33 (0.59–9.14)	1.95 (0.52–7.33)
**Explanatory variables**				
Time in university hospital at rank		0.95 (0.88–1.04)		
Time in university hospital at rank				
1–2 years			0.43 (0.17–1.07)	
3–4 years			4.02* (1.12–14.48)	
5–6 years			1.06 (0.28–4.05)	
> = 7 years			0.27* (0.08–0.91)	
University hospital yes/no (previous year)				0.09* (0.01–0.78)
Interaction^3^				11.18* (1.29–96.60)
**Observations**		2,971	2,971	2,971
**Pseudo R-squared**		0.570	0.577	0.576
**Log likelihood**		-288.2	-283.9	-284.6

^1^Reference category = internal medicine.

^2^Reference category = female.

^3^Interaction of *inter-organizational move* and *university hospital yes/no (previous year).*

*** p <0.001; ** p <0.01; * p <0.05.

#### Promotion from junior physician to senior physician

Three regression models were estimated to analyse the impact of working in a university hospital on the chance of promotion to a senior physician position. Model 1 indicates no significant connection between the cumulated time spent in university hospitals as a junior physician and the probability of reaching a senior physician position. In Model 2, the variable for working in university hospitals for 1–2 years is significant (OR = 0.38, p < 0.01). Physicians working in a university hospital for 1–2 years have a 62% smaller chance of becoming a senior physician, whereas working in a university hospital for 3–4, 5–6, or > =7 years neither increases nor decreases the chance of promotion to a senior physician position. With Model 3 we examined the impact of working in a university hospital in the year previous to the promotion and the interaction between this variable and changes between hospitals on the chance of promotion. The coefficients are not significant.

#### Promotion from senior physician to chief physician

Three regression models were examined to analyse the influence of working in university hospitals on the promotion success of senior physicians moving to a chief physician position. The regression model with the explanatory variable *time in university hospital as senior physician* (Model 1) was not significant. Model 2, with the split time variables for years spent in university hospitals, indicates a positive influence on promotion to a chief physician position for 3–4 years spent in university hospitals (OR = 4.02, p < 0.05) and a negative impact on the chance of promotion to a chief physician position for those spending > =7 years (OR = 0.27, p < 0.05) in university hospitals. Conversely, we identified no effect on the chance of promotion for working in university hospitals for 1–2 years (OR = 0.43, p < 0.10) or 5–6 years (OR = 1.06, p > 0.10). Inspection of the coefficient for being in a university hospital in the year preceding a promotion (OR = 0.09, p < 0.05) indicates that there is nearly no chance of promotion for a physician remaining in the same university hospital, i.e. in this instance there is no promotion without mobility. However, the corresponding interaction effect of this variable and an inter-organizational move between hospitals has a high positive effect on the chance of promotion (OR = 11.18, p < 0.05). The interaction effect shows that a change from a university hospital increases the chance of promotion to a chief physician position more than a change from a hospital without university status.

## Discussion

The results of this quantitative longitudinal study prove that working in university hospitals has an impact on the careers of clinical physicians. It is apparent that the impact of working in a university environment on the duration of promotion and promotion success varies according to the career stage.

### Promotion duration

One career phase considered was the time period spent as a junior physician leading up to promotion to senior physician. In this phase, physicians who spent a part of their time as a junior physician in a university hospital took a longer time to reach the next promotion step than a junior physician who spent no time in university hospitals. Reasons for this result cannot be explained by the present study. Studies into the workplace experience of junior physicians in German-speaking Switzerland show that junior physicians in university hospitals and large-scale hospitals have significantly greater concerns about insufficient supervision by experienced colleagues than junior physicians at smaller institutions. Furthermore, junior physicians at university hospitals and large-scale hospitals report significantly worse workplace conditions and a greater imbalance between efforts and rewards [29,30]. Similar working conditions for junior physicians in training may also exist in German university hospitals as the medical services in Switzerland and Germany have similar career structures. Further research is needed to confirm or reject this.

In the career phase from senior physician to chief physician the result differed from that seen in the earlier career phase. Senior physicians working in university hospitals for the entire time period spent in this rank reached chief physician position faster than physicians who had not spent the entire time in university hospitals. For a senior physician looking to attain a chief physician position the features of university hospitals appear to be conducive to career advancement, potentially as a result of the scope the services and treatments offered and the complexity of the organization.

### Promotion success

The analysis of the promotion success for promotion from junior physician to senior physician and senior physician to chief physician also emphasizes that working in university hospitals has career-enhancing effects in the career phase leading up to promotion to a chief physician position. For senior physicians working in a university environment for a medium time the chance of promotion to a chief physician post increases significantly. Conversely, working in university hospitals for a short time span during the career phase as a junior physician has a negative impact on promotion to a senior physician position, and for medium or long time spans no evidence of a positive or negative effect on promotion was found. However, working in university hospitals on the career trajectory to a chief physician position has a negative effect on promotion chances if the physician remains in university hospitals longer than a certain time. This effect, where the chance of promotion decreases after a certain firm tenure or age of an employee, is not only specific to the profession of physicians but is also evident in other professions [31]. Evidence has been found that physicians from university hospitals are the preferred choice when hiring to chief physician positions, but not for when hiring to senior physician positions.

### Limitations

Owing to the origin of the dataset, self-selection effects relating to a sample which contains mainly the histories of career-orientated physicians cannot be ruled out completely. Despite this possible bias we opted to use this database as almost all chief physician posts in Germany are filled with external candidates, either through job advertisements or with the help of a consultancy firm. Furthermore, the study design, with its discrete-time event history analysis, requires data from physicians with ambitions to advance to a higher position, since only these physicians are at risk - or in this context, have the chance - of reaching a higher position.

## Conclusions

This study indicates that the career success of clinical physicians is influenced by the extent to which they work in university hospitals. In this way, different effects were found for junior and senior physicians looking to move up to the next hierarchical career level. The investigation of the promotion duration shows that junior physicians with some time spent working in university hospitals take longer to attain promotion to a senior physician position than physicians without work experience in a university hospital. However, senior physicians that spent the entire time period leading up to promotion in university hospitals gained a chief physician position faster than colleagues who had spent only some or none of this time in university hospitals. The analysis of promotion success also suggests that a medium time period spent working in university hospitals has a positive impact on the promotion chances of senior physicians, while the promotion chances of junior physicians neither increase nor decrease on the same terms. Furthermore, the probability of rank advancement for senior physicians changing from a university hospital is positive, while for a junior physician a change from a university hospital has no effect.

## Competing interests

The authors declare that they have no competing interests.

## Authors’ contributions

Both authors participated in the conception and design of the study. Acquisition of data, data analysis, interpretation of data, writing of the manuscript and final approval of the manuscript to be published was made by CD. LK contributed methodological advice and critically revised the drafts of the paper. Both authors read and approved the final manuscript.

## Pre-publication history

The pre-publication history for this paper can be accessed here:

http://www.biomedcentral.com/1472-6920/14/85/prepub

## Supplementary Material

Additional file 1 Survival curves for years from junior to senior physician in university hospitals. Click here for file

Additional file 2 Survival curves for years from senior to chief physician in university hospitals. Click here for file
