# Supplementary material for: University hospitals as drivers of career success: an empirical study of the duration of promotion and promotion success of hospital physicians
Source: BMC Med Educ. 2014 Apr 23;14:85. doi: 10.1186/1472-6920-14-85 (PMC4021192; doi:10.1186/1472-6920-14-85)

Additional file 1: Survival curves for years from junior to senior physician differentiated by >=1, >=3, >=5 and >=7 years in university hospitals

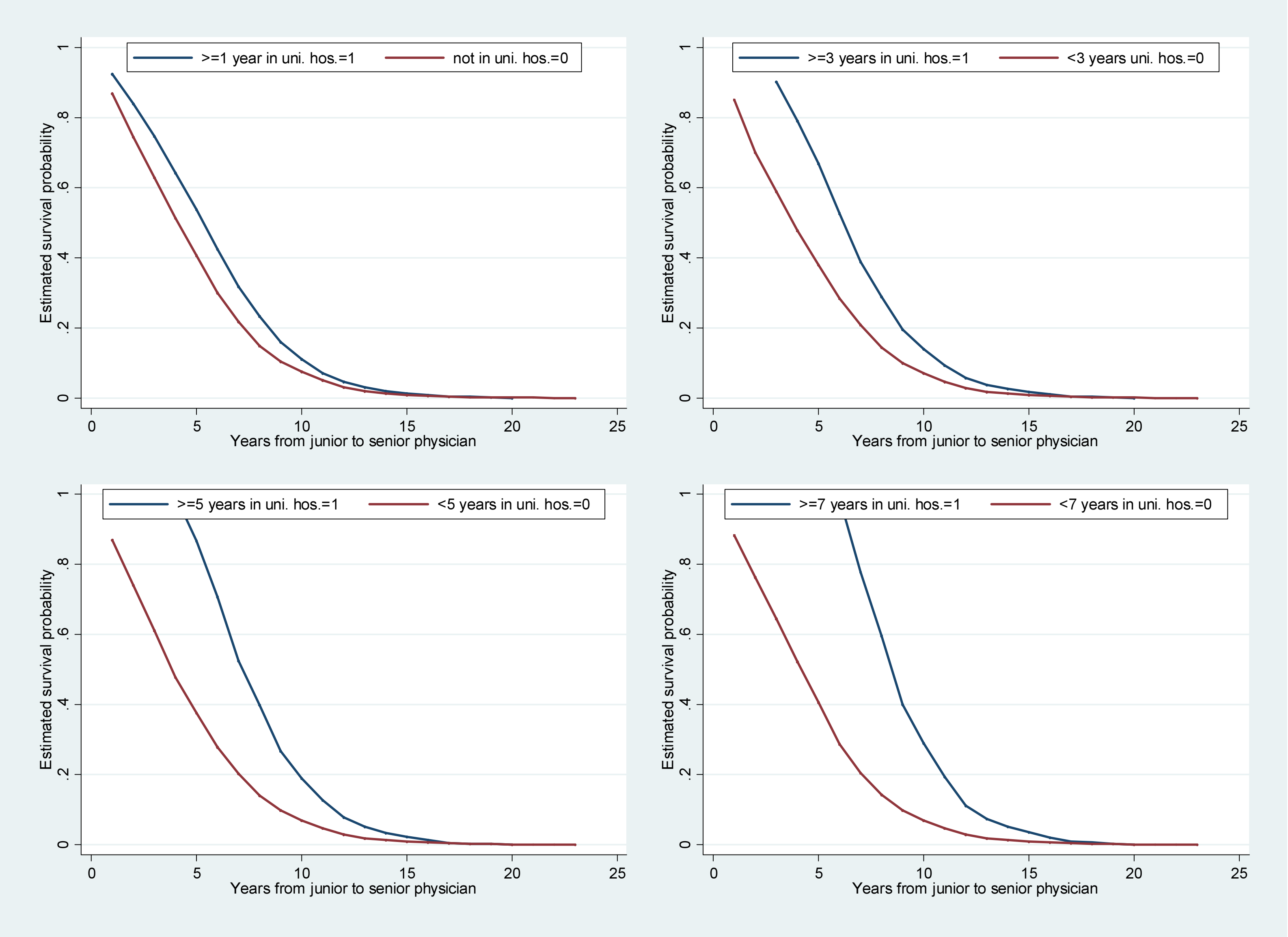

Supplement: Additional file 1 — Survival curves for years from junior to senior physician in university hospitals. [file 1472-6920-14-85-S1.pdf]
